# Coiled distal internal carotid artery (ICA) aneurysm in transcranial sonography

**Published:** 2017-07-06

**Authors:** Masoud Mehrpour, Fahimeh Haji-Akhoundi, Babak Zamani

**Affiliations:** Department of Neurology, Firoozgar Hospital, Iran University of Medical Sciences, Tehran, Iran

**Keywords:** Doppler Transcranial Sonography, Aneurysm, Internal Carotid Artery

A 45-year-old man presented with a thunderclap headache. Brain computed tomography (CT) scan without contrast showed massive subarachnoid hemorrhage (SAH). We transferred him to the angiography unit where he was diagnosed with a left (Lt) distal internal carotid artery (ICA) aneurysm. We secured the aneurysm using 8 coils. He was stable the following days. 

**Figure 1:**
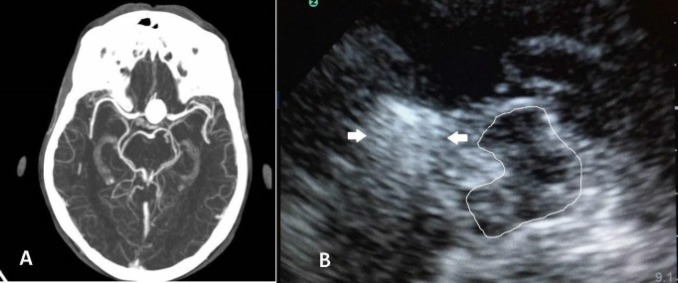
A. Axial brain computed tomography (CT)-angiography showing a giant left (Lt) distal internal carotid artery (ICA) aneurysm. B. Axial transcranial sonogram (mesencephalic level): Butterfly-shaped midbrain was encircled for better visualization (solid lines). Arrows indicate the coiled distal ICA aneurysm.

He was monitored via daily transcranial sonography (TCS) for the potential development of vasospasm. In TCS, the coiled distal ICA aneurysm was visualized as a round hyperechoic mass anterior to the midbrain. There was no aneurysm refilling on consequent evaluations. Unenhanced TCS is proposed as a screening tool for coiled aneurysm refilling.^1^

**Figure 2:**
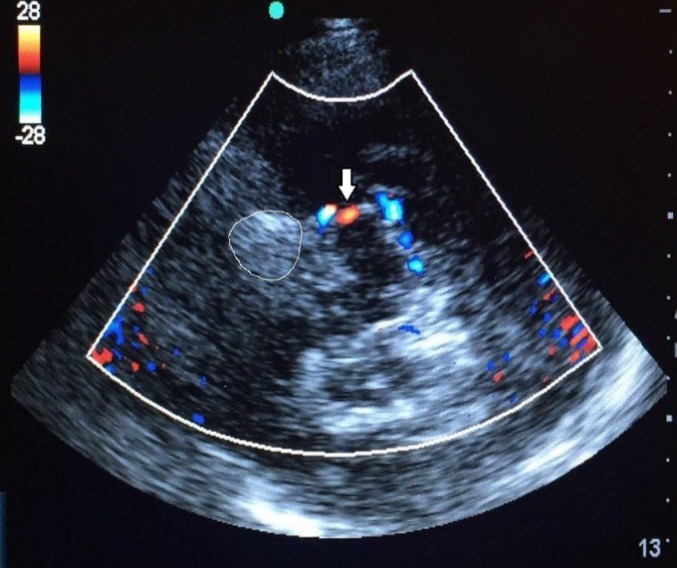
Axial transcranial color sonography (mesencephalic level); coiled aneurysm was encircled. There was no refilling within the aneurysm. Arrow indicates posterior cerebral artery (PCA).

## Notes:

**How to cite this article:** Mehrpour M, Haji-Akhoundi F, Zamani B. Coiled distal internal carotid artery (ICA) aneurysm in transcranial sonography. Iran J Neurol 2017; 16(3): 162-3.
